# Supplementary figures and images for: Classification of ovarian cancer associated with BRCA1 mutations, immune checkpoints, and tumor microenvironment based on immunogenomic profiling
Source: PeerJ. 2020 Nov 24;8:e10414. doi: 10.7717/peerj.10414 (PMC7694562; doi:10.7717/peerj.10414)

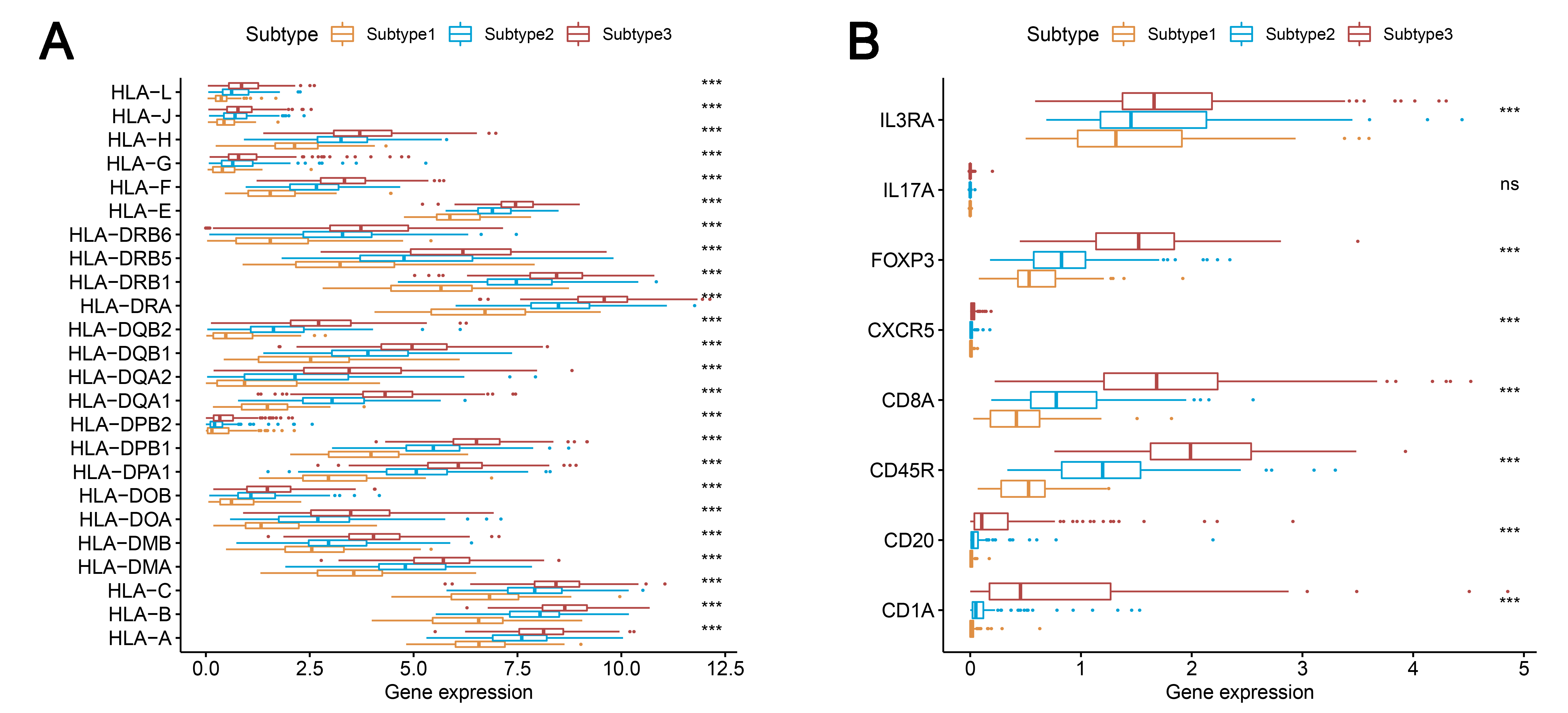

Supplement: Figure S1 — (A) Comparison of the expression levels of the HLA genes among the three subtypes. (B) Expression levels of the immune cell subgroup maker genes among the three subtypes. HLA, human leukocyte antigen. *** P < 0.001. TCGA, The Cancer Genome Atlas dataset [file peerj-08-10414-s006.png]

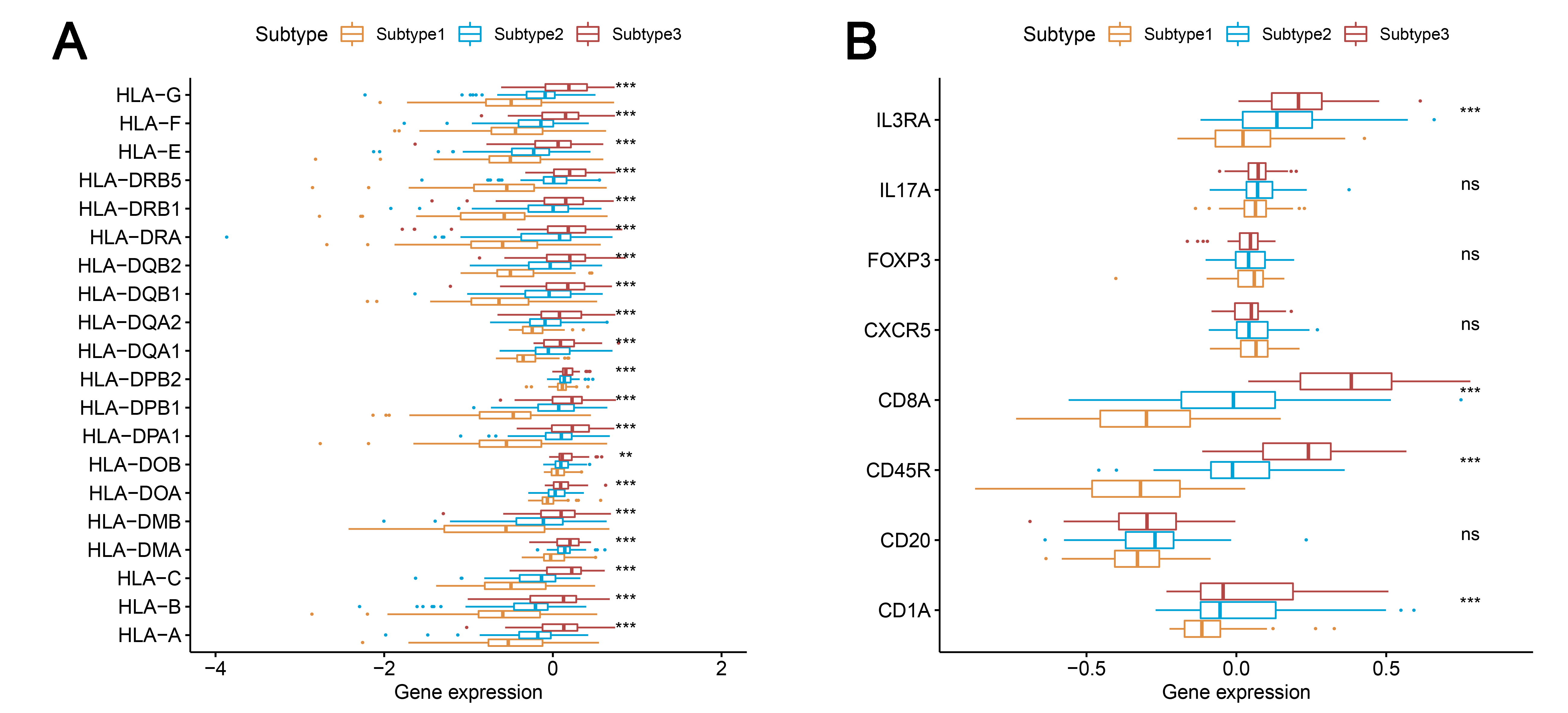

Supplement: Figure S2 — (A) Comparison of the expression levels of the HLA genes among the three subtypes in the GEO dataset. (B) Expression levels of the immune cell subgroup maker genes among the three subtypes in the GEO dataset. * P < 0.05, ** P < 0.01, *** P < 0.001. GEO, Gene Expression Omnibus. [file peerj-08-10414-s007.png]
